# COVID-19 Prevention Measures Implemented by Tennis Coaches: The Role of Continent, Experience, and Type of Facility

**DOI:** 10.3390/ijerph182312679

**Published:** 2021-12-01

**Authors:** Rafael Martínez-Gallego, Juan Pedro Fuentes-García, Miguel Crespo

**Affiliations:** 1 Department of Sport and Physical Education, University of Valencia, 46010 Valencia, Spain; Rafael.martinez-gallego@uv.es; 2 Didactic and Behavioral Analysis of Sports Research Group (ADICODE), Faculty of Sport Sciences, University of Extremadura, 10003 Cáceres, Spain; 3 Development Department, International Tennis Federation, London SW15 5XZ, UK; Miguel.Crespo@itftennis.com

**Keywords:** racquet sports, tennis, coaches, COVID-19

## Abstract

The prevention strategies used by tennis coaches when delivering tennis lessons during the COVID-19 pandemic were analyzed in this study. An ad hoc questionnaire collected data from 655 Spanish and Portuguese speaking tennis coaches working in Latin America and Europe. Differences in the prevention measures were analyzed according to the continent, the coaches’ experience, and the type of facility they worked in. Results showed that coaches used information provided from local and national organizations more than from international ones. Hand hygiene, communication of preventive strategies, and changes in the coaching methodology were the most used prevention measures. Latin American coaches and those working in public facilities implemented the measures more often than their European colleagues or those working in private venues. Finally, more experienced coaches showed a greater awareness of the adoption of the measures than their less experienced counterparts. The data provided by this research may assist in developing new specific guidelines, protocols, and interventions to help better understand the daily delivery of tennis coaching in this challenging context.

## 1. Introduction

The coronavirus disease (COVID-19) caused by severe acute respiratory syndrome-coronavirus-2 (SARS-CoV-2) infection leads to still unknown and unusual health conditions that are challenging to manage [1,2]. In this context, sport and exercise programs and adequate physical activity levels have been shown to be well-known modulators of the clinical manifestations and prognosis in many chronic diseases. Sport coaches are the main deliverers of these programs, which are geared to individuals of all ages, genders, physical and mental conditions, and skill levels [3].

Governments and different organizations at their various levels of responsibility have determined and implemented social distance as well as health and safety measures to ensure a secure practice of these sports activities [4,5]. In the specific case of tennis, the International Tennis Federation (ITF), the world governing body of tennis, published the Return to Tennis guidelines [6], in which it was stated that: “The primary aim of this document is to set guidelines for minimum and recommended standards for the organization of tennis competitions. This document has been developed in accordance with the WHO guidelines on the organization of sports events and with input from the Chair of the ITF Sport Science & Medicine Commission. It is intended to supplement the existing organizational requirements for ITF events, rather than a substitute for them” (p. 12).

Authors such as Crespo and Jabaloyes [7] have reflected on the new aspects that will have to be considered in tennis coaching during and after the pandemic. These authors have indicated that the changes the pandemic has generated for coaches have required a crucial adaptation in the delivery of programs to athletes and engagement with those involved in sports activities. The challenges to plan content, evaluate performance, prevent injury, and facilitate learning should be addressed in a creative and flexible way while ensuring the necessary health and safety measures. For instance, measures relating to equipment, methodology, and communication may be adopted at the discretion of coaches [8,9].

They play a key role in delivering the sport by teaching players, organizing competitions, administering programs, running events, and managing venues, among other functions. It is estimated that there are around 165,000 tennis coaches worldwide, with close to 20% being women [10]. The global COVID-19 outbreak has had a significant impact on tennis coaching. Movement restrictions imposed by governments have resulted in the suspension of the delivery of tennis programs at all levels of the game, from club sessions to high-performance training in academies. The restrictions imposed by the relevant authorities have been changing and adapting to the evolution of the pandemic in the different countries. When these have been relaxed, it has been possible to organize coaching sessions and programs with fewer players per court and following the set guidelines. When they have been enforced, no coaching has been allowed at all, and in some cases, only international competitions with no spectators have been organized.

As per the impact of COVID-19 in tennis, since March 2020 the tennis ecosystem has been living in uncertain and unprecedented times. This has had an obvious impact on tennis stakeholders. During this period, the major tennis organizations—the ITF, Tours and Grand Slams—as well as other national associations have provided financial and material assistance to players, member nations, officials, and coaches, which has been widely welcomed. Many of them have put forward initiatives to provide guidance to stakeholders on how to face the pandemic. As an example, the United States Tennis Association put forth some financial aid and significant guidance for teaching professionals and recreational clubs to guide communities to a safe return to tennis once local restrictions are lifted [11]. Other associations and federations, such as the Lawn Tennis Association (LTA), the French Tennis Federation (FFT), Tennis Australia (TA), the German Tennis Federation (DTB), the Royal Spanish Tennis Federation (RFET), and the Italian Tennis Federation (FIT), to name a few, have also published guidelines, protocols, and regulations adapted to the scenarios in each of their jurisdictions (for a summary, see Santilli and Crespo [9].

From a research perspective, several studies have dealt with COVID-19 and tennis. King [12] studied the response of the professional tennis ecosystem, mostly the ATP and the WTA. The gender gap in the propensity to voluntarily withdraw because of COVID-19 concerns among players who were eligible and fit to play the 2020 US Open was analyzed by Kowalik and Lewandowski [13]. In the college tennis environment, talent migration of International Sport Athletes in fv Division I tennis was explored by Parrish, Otto, and Dodson [14]. The adaptation and destabilization on interpersonal relationships between tennis players and coaches during the lockdown was studied by Antonini et al. [15]. Bonavolontà et al. [16] evaluated the levels of tennis play, enjoyment, and motivation in children and adolescents in the emergency context. The interaction between tennis players and coaches during the pandemic through a Learning Management System that allowed teaching the game without physically practicing on-court was analyzed by Rahman et al. [17]. Furthermore, several book chapters have also covered the impact of COVID-19 on the tennis ecosystem. Bradbury and Galloway [18] discussed the perspectives in New Zealand tennis, and Slater and Watkins [19] analyzed tennis players’ responses to the global pandemic’s impact on professional tennis governance. 

In a recent study, Crespo et al. [20] investigated the perceptions of Spanish- and Portuguese-speaking tennis coaches working in Latin American and European countries with regard to the impacts of the COVID-19 pandemic on their health, professional, and economic circumstances. Coaches reported on the incidence of the virus in terms of infection and quarantine as well as the impact on their coaching programs, professional development, training, and education. They were also asked about their perception of the overall situation as a threat. The results showed that the COVID-19 pandemic has had a considerable impact on the health and the profession of tennis coaches. Although Latin American coaches reported a greater impact on their health, economic, and professional circumstances, they viewed the pandemic as an opportunity for professional improvement and training as compared to the perceptions of European coaches.

As can be noticed from the studies above, there are several studies that have covered the topic of COVID-19 and tennis by investigating a few contexts within the tennis ecosystem. However, those related to tennis coaches are scarce, and to the authors’ knowledge, no study has investigated the views of sport and tennis coaches on the guidelines they have had to adopt to carry out their work in the context of the pandemic and the organizations that dictate these guidelines. More specifically, the research question of this study is to find out the coaches’ perceptions on the implementation of social distancing, health, security, and hygiene measures, the adaptation of equipment, the variations to the teaching methodology, and the content and sources of communication.

It was believed that identifying coaches’ perceptions of lockdown and the prevention strategies they used when delivering tennis lessons could be useful for the development of new specific guidelines, protocols, and interventions to help better understand the daily delivery of tennis coaching. Whilst this could be done on a general level, examination of tennis coaches’ specific behaviors and experiences could assist in developing interventions based in real world experiences. Therefore, it was hypothesized that local authorities would influence coaches more than national or international ones when implementing the guidelines, that more experienced coaches would implement more protocols than their less experienced counterparts, and that there would be no differences in the application of these guidelines as related to the type of venue the coaches were working in or where they would be based (in Europe or America).

## 2. Materials and Methods

### 2.1. Study Design

The study was a quantitative descriptive cross-sectional study based on a survey methodology [21].

### 2.2. Sample

A total of 655 Spanish and Portuguese speaking tennis coaches working in Latin American (n = 219) and European (n = 436) countries took part in the study. The selection criteria for the research included geographical (working in Latin American or European countries) and linguistic (Spanish or Portuguese speaking). Therefore, coaches who were working in other continents or spoke other languages were excluded from the study. The gender breakdown of the sample was 88% (n = 578) men and 12% (n = 77) women. In terms of coaching experience, 25% (n = 162) had up to 10 years of experience, and 75% (n = 493) had more than 10 years of experience. Regarding the venue they were working at, 29% (n = 188) worked in public venues and 71% (n = 467) in private facilities. In terms of their commitment to coaching, 70% (n = 457) worked full time, while 30% (n = 198) worked part time.

### 2.3. Instrument

Data was collected using an ad hoc questionnaire designed and validated for the study in four stages. An initial review of the relevant literature and the appropriate information available from the different tennis organizations was conducted to generate the main variables as well as adequate items and their definitions. This was followed by a discussion among the authors to reach an agreement on the selected items, their definitions, and an initial draft of the questionnaire. The following step consisted of a quantitative and qualitative evaluation made by 5 tennis managers, coaches, and coach educators, who analyzed, evaluated, and suggested changes to the definitions and the items proposed. Based on these improvements, a final version of the instrument was designed. The 18-item questionnaire was divided into four sections: (1) social distance; (2) health, security, and hygiene; (3) equipment and methodology; and (4) communication (Table 1).

### 2.4. Procedure

Data collection lasted for 31 days (from 20 January to 19 February 2021), with the number of daily infections in the world in the middle of the data collection, 3 February 2021, at 488,329, and the number of deaths at 15,477 [22]; the number of people having contracted the disease was more than 103,827,020, of which at least 63,195,000 were recovered, with at least 2,253,813 deaths in the world. To this date, Europe had 751,095 deaths (33,794,433 infections) and Latin America and the Caribbean, 602,222 deaths (19,085,319 infections) (Johns Hopkins University, 2021). Thus, as of 3 February 2021, considering the aforementioned data and the population under study, based on the estimated number of inhabitants [23], Europe had 739,673,422 inhabitants, with 0.10% of deaths and 4.568% of infections, while Latin America and the Caribbean had 670,124,155 inhabitants, with 0.09% of deaths and 2.848% of infections. Convenience and snowball sampling techniques were used to recruit the tennis coaches. With the support and assistance of the Participation & Education Area of the International Tennis Federation, an online survey, a reliable tool that provides advantages for both participants and researchers [24], was placed on various social media platforms. Furthermore, due to the authors extended relationships with tennis coaches worldwide, the survey was sent to the Directors of Coaches Education Departments of the Spanish and Portuguese speaking National Associations for them to share with the coaches of their respective countries. Moreover, the researchers followed a snowball sampling process, directly inviting coaches who were part of their personal and professional circle and asking them to extend the invitation to their colleagues [25]. At the start of the online survey, the coaches were shown a description of the study and were informed about the confidentiality and voluntary nature of the research, in accordance with the Declaration of Helsinki. All the procedures were approved by the Commission of Bioethics and Biosecurity of the University (approval number: 57/2020).

### 2.5. Data Analysis

The data obtained through the online questionnaire were exported to the Microsoft Excel program, where the data were processed to format them and make them suitable for analysis. Data analysis was carried out using the SPSS v26 statistical package. The Kolmogorov-Smirnov test was performed to check whether the variables were normally distributed. When deviations from normality were found for all variables, nonparametric statistics were used, employing the Chi-square test to analyze the differences between groups. Effect size was measured using Cramer’s V (0.10 small; 0.30 medium; 0.50 large). The significance level was set at *p* < 0.05.

## 3. Results

**Figure 1:**
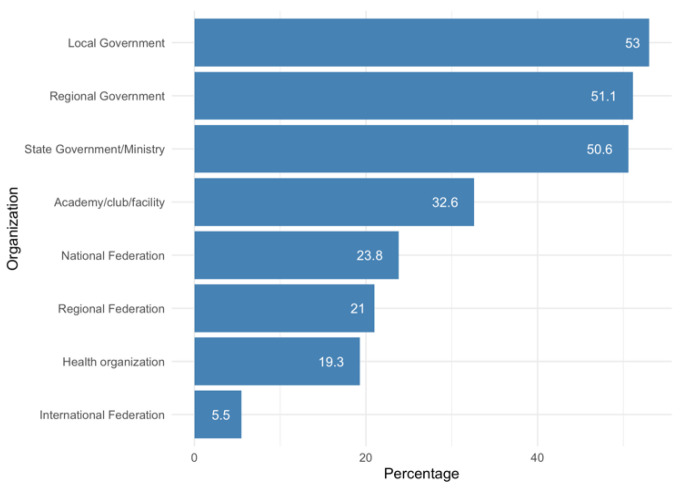
Organizations imposing measures on tennis coaches.

Figure 1 shows the organizations that have imposed measures on tennis coaches. As can be seen, the most important organizations in terms of regulating the activity with regards to COVID-19 measures taken were mainly local, regional, and state governments. Each of these three types of organizations imposed measures on more than 50% of the coaches surveyed. By contrast, the organization that played a lesser role in terms of measures imposed on coaches was the International Tennis Federation. Only 5.5% of coaches indicated that this organization imposed some kind of COVID-19–related measures on them.

**Figure 2:**
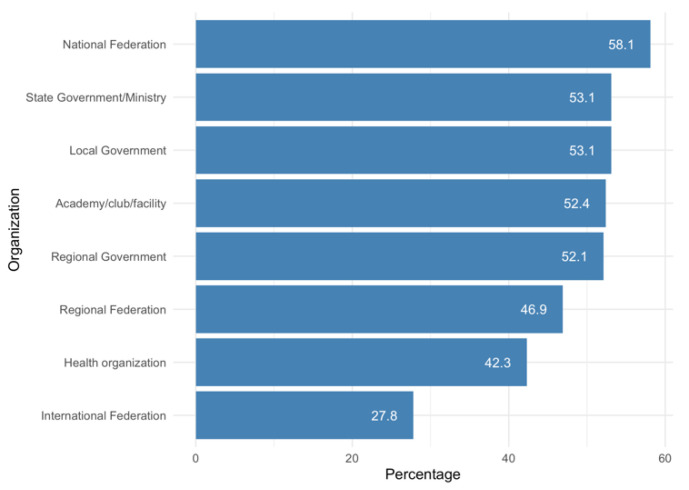
Sources of information consulted by coaches.

Regarding the institutions or organizations that served as a source of information for the coaches on COVID-19–related actions to be taken, the National Federations were the most consulted by the coaches, while the International Federation was the least consulted (Figure 2).

Table 2 shows the percentage of coaches who adopted the measures for each of the categories. The measures adopted by the highest percentage of coaches, with values above 96%, were hand disinfection and communication of COVID-19–related measures. It is also remarkable that 94.5% of coaches took measures related to drills and coaching methodology. The least used measures were the use of different balls for each player (15.1%) and the age-related access restrictions (34.4%). In terms of differences according to continent, significant differences were found in all items except hand disinfection, adapting exercises and coaching methodology, and player registration. Except for the use of masks by the coach and the players, which was more prevalent in coaches in Europe, in the rest of the measures, the percentage of coaches who adopted them was higher in Latin America. The items that showed the largest effect sizes were the reduction of the number of players, the age-based access restrictions, the temperature measurement, and the use of different balls for each player.

Measures related to social distance and health, security, and hygiene are more general measures often imposed by governments or organizations. However, the measures concerning equipment and coaching methodology used and communication strategies are more specific and depend to a greater extent on the decisions of the coaches themselves. Therefore, it was decided to compare these types of measures depending on the experience of the coaches and the type of facility in which they worked. 

Table 3 shows the differences in the percentages of coaches who used measures related to equipment and methodology, as well as communication, according to their experience. As can be seen, more experienced coaches showed higher percentages than their less experienced counterparts in terms of adopting all measures, except for restrictions on sharing equipment, adaptations of exercises and methodology, and consent to participate in classes, where no significant differences appeared. The largest effect sizes were found for the items related to the disinfection of equipment and the use of different balls for each player.

As per the differences according to the type of facility in which the coaches carried out their work, differences were found only in the items related to the disinfection of the equipment and the registration of players. Both measures were adopted to a greater extent by coaches working in public facilities (Table 4).

## 4. Discussion

This article analyzed the measures and strategies adopted by tennis coaches during their tennis lessons for the prevention of COVID-19. Furthermore, the organizations that imposed measures and the main sources of information that coaches made use of were analyzed. Coaches were asked about the measures taken in terms of social distance, health, security, hygiene, equipment, methodology, and communication. In addition, differences in the implementation of these measures were analyzed according to the continent, the coaches’ experience, and the type of facility in which they were working.

The results of our study show the measures imposed on coaches by different organizations. Sporting activities such as tennis are part of everyday life in society, so it is logical that local, regional, and state governments play a greater role in regulating the activity of tennis coaches than international organizations. On the other hand, regarding the sources that coaches use to obtain information, the results show that national federations play a fundamental role. Numerous federations from different countries published documents with specific recommendations and guidelines for tennis players and coaches that complemented the general measures imposed by governments [26,27,28,29]. These recommendations, being specific to tennis, presented aspects that were much more applicable to the situations that coaches deal with in their professional work on the court. This is why the local and national federations were the main source of information for tennis coaches.

Hand hygiene is one of the measures that was promoted the most by the different organizations in charge of preventing the transmission of the COVID-19 virus among the population. In fact, the WHO carried out campaigns aimed at this aspect, indicating that hand hygiene is one of the most effective actions to reduce the spread of the COVID-19 virus [30]. This seems to be the main justification for why this measure was the most widely used by tennis coaches. Another measure, which was adopted by more than 96% of the coaches, was communication to the players of the actions to be carried out related to COVID-19 prevention. It seems clear that this aspect plays a key role in meeting the established standards. It is difficult to comply with the measures to be taken if they are not communicated efficiently to the people who must comply with them. In addition, from a stress response perspective, it has been shown that clear communication with players and their involvement in the management of guidelines and preventive measures can facilitate an adaptive response to stress [31]. On the other hand, tennis, being a non-contact sport in which the distance between players is considerable as compared to other team or contact sports, has been shown to be one of the safest sports in terms of preventing COVID-19 transmission [7]. However, during tennis lessons, it is common that the number of players per court increases, and therefore the distance between players is reduced and the chances of contact increase. In this context, to increase the distance between players and avoid contact, it is reasonable that a large percentage of coaches indicated that they adapted the methodologies and exercises used during training sessions. To address this scenario, several authors and organizations published methodological proposals and examples of drills and exercises that helped coaches to organize lessons in a safe environment for players [32,33].

Regarding the differences between continents, in almost all measures, the percentage of coaches who carried them out was higher in Latin America than in Europe. These differences were particularly important in the reduction of the number of players, the age restrictions on access, the temperature measurement, and the use of different balls for each player, which were the variables where the largest effect sizes were obtained. Research has shown that Latin American tennis coaches suffered a greater impact from a health, economic, and professional point of view as compared to their European counterparts; however they have seen the pandemic as an opportunity for professional improvement and professional education as compared to European coaches [20]. This may be the reason why there was a greater awareness on the part of Latin American coaches than European coaches in the application of prevention measures. However, it is very important to highlight that mask use was less widespread among Latin American coaches and players than among the European contingent of the sample, and even more so when the governments of the countries in both regions recommended mask use as of March 2020 [34]. One explanation could be that the Latin American countries were, in general, not self-sufficient to ensure an adequate availability of masks and, moreover, did not have previous experience in the use of masks. These facts may explain to some extent the results of this study related to the use of masks.

On the other hand, some previous studies have looked at stress levels in sports coaches, reporting that coaches with less experience showed higher levels of stress due to the COVID-19 pandemic [35,36]. In the case of tennis coaches, this aspect does not seem to be related to the measures adopted by the coaches since, according to the results obtained in this study, it was the more experienced coaches who showed a greater awareness of the adoption of measures related to equipment, methodology, and communication, especially measures related to the disinfection of equipment and the use of different balls for each player. Studies that have sampled the general population have shown that compliance with COVID-19-related measures is lower in younger adults than in older adults [37], so the results obtained in this study confirm that this is also the case in the population of tennis coaches.

As per the type of facility in which the coaches conduct their classes, differences were found only in the items relating to the disinfection of equipment and the registration of players. It seems that this variable does not have a great influence on the measures taken by the coaches. The two variables in which differences appeared, the disinfection of the equipment and the registration of players, seem to be related to the fact that, on many occasions, in public facilities, the number of users is higher, and the space is shared with other sport disciplines and therefore with other athletes [38]. This fact makes the disinfection of the equipment more important, as it is shared with a greater number of people, and demands adequate registration of players, as this is an essential requirement for the facility to be able to manage information properly.

## 5. Conclusions

This article analyzed the views of tennis coaches in terms of the organizations that provided information and guidelines for the prevention of COVID-19 as well as the measures and strategies adopted by them during the delivery of their tennis lessons.

Even though this study followed the objectives and methods used in previous research, there are several limitations that can be identified, such as the sample characteristics (i.e., coaches from other regions) and the fact that coach gender could have been considered as a variable. Future research lines include the study of the views of coaches about the return to tennis activities as the restrictions are lifted in different countries worldwide. Other research topics could include the study of the behaviors of coaches from other regions or a comparison between coaching methods and practices depending on the level of the players.

The results showed that coaches perceived that they received and used more information and guidelines provided from local and national organizations than from international ones. Furthermore, measures such as hand hygiene, communication of preventive strategies, and changes in their coaching methodology and instruction were the most used ones to address the challenge created by the pandemic. Results also showed that coaches working in public facilities implemented the measures more often than their colleagues working in private venues and that Latin American coaches used many of these measures more often that their European counterparts. Finally, more experienced coaches showed a greater awareness of the adoption of measures related to equipment, methodology, and communication, especially measures related to the disinfection of equipment and the use of different balls for each player.

This study contributed to a better understanding of the views and behaviors of tennis coaches in terms of the guidelines they had to carry out in the context of the pandemic and the organizations that dictated these guidelines. By identifying the coaches’ perceptions in lockdown and the prevention strategies they used when delivering tennis lessons, tennis organizations can develop new specific guidelines, protocols, and interventions to help better understand the daily delivery of tennis coaching. 

## Figures and Tables

**Table 1:** Categories and sub-categories included in the survey of the study.

Category	Sub-Category	Item
Social distance	Player reduction	Have you been forced to reduce the number of players per court in your lessons?
	Companion reduction	Is the number of people accompanying players to the facility during lessons limited?
	Age restrictions	Is attendance at tennis lessons limited to players of any age range?
	Group restrictions	Has player interaction with players from other groups/classes been restricted?
	Time restrictions	Have restrictions been adopted regarding the time spent in the facility (e.g., 15 min before and after the lesson)?
Health, security, and hygiene	Temperature	Have players’ temperatures been taken or are they taken before entering lessons?
	Hand cleaning	Have you and your players disinfected or do you disinfect your hands before entering the lesson?
	Coach mask	Have you used or do you use a face mask or other protective equipment during the lessons?
	Player mask	Have the players used or do they use face masks or other protective equipment during the lessons?
	Protocol	Is there a protocol in place in case of detecting symptoms of a player during lessons?
Equipment and methodology	Shared equipment restrictions	Have you avoided or do you avoid the use of shared material during lessons?
	Equipment disinfection	Have you disinfected the equipment before each lesson?
	Different balls	Has each player used or does each player use different balls?
	Drills/Methodology adaptations	Have you adapted or do you adapt exercises/methodology to maintain social distance, use less material, etc.?
Communication	Measures	Have you informed your players, in advance, of the hygiene measures required to attend tennis lessons?
	Players’ record	Do you have a detailed record of your players’ contact details so that they can be contacted in case of a positive case?
	Consent	Have you sought consent from parents/guardians of players under the age of 18 for their participation in tennis lessons?
	Online payment and bookings	Has a system of online payment and booking of lessons been established due to COVID-19?

**Table 2:** Percentage of coaches who carried out the measurements for each of the categories according to the continents.

Category	Item	Total (n = 655)	Latin America (n = 219)	Europe (n = 436)		
		%	%	%	*p*	ES
Social distance	Player reduction	76.0	90.9	68.6	<0.01 **	0.246
	Companion reduction	88.5	92.2	86.7	<0.05 *	0.082
	Age restrictions	34.4	63.5	19.7	<0.01 **	0.435
	Group restrictions	75.3	80.4	72.7	<0.05 *	0.084
	Time restrictions	82.6	88.1	79.8	<0.01 **	0.103
Health, security, and hygiene	Temperature	69.8	84.0	62.6	<0.01 **	0.220
	Hand cleaning	96.8	96.8	96.8	1	0
	Coach mask	83.5	76.3	87.2	<0.01 **	0.139
	Player mask	51.5	44.3	55.0	<0.01 **	0.102
	Protocol	85.3	87.2	84.4	0.34	0.04
Equipment and methodology	Shared equipment restrictions	85.8	90.0	83.7	<0.05 *	0.084
	Equipment disinfection	74.5	84.9	69.3	<0.01 **	0.170
	Different balls	15.1	29.2	8.0	<0.01 **	0.279
	Drills/Methodology adaptations	94.5	96.3	93.6	0.14	0.057
Communication	Measures	96.6	98.6	95.6	<0.05 *	0.078
	Player record	85.8	87.7	84.9	0.33	0.038
	Consent	75.4	82.2	72.0	<0.01 **	0.111
	Online payment and bookings	60.2	68.9	55.7	<0.01 **	0.127

ES = effect size; ** Significant differences (*p* < 0.01); * Significant differences (*p* < 0.05).

**Table 3:** Percentage of coaches who implemented the equipment and coaching methodology measures and communication according to their experience.

Category	Item	Total (n = 655)	Up to 10 Years of Experience (n = 162)	More Than 10 Years of Experience (n = 493)		
		%	%	%	*p*	ES
Equipment and methodology	Shared equipment restrictions	85.8	84.0	86.4	0.44	0.030
	Equipment disinfection	74.5	65.4	77.5	<0.01 **	0.119
	Different balls	15.1	7.4	17.6	<0.01 **	0.123
	Drills/Methodology adaptations	94.5	92.0	95.3	0.10	0.064
Communication	Measures	96.6	93.8	97.6	<0.05 *	0.090
	Players’ record	85.8	80.2	87.6	<0.05 *	0.091
	Consent	75.4	75.3	75.5	0.97	0.001
	Online payment and bookings	60.2	53.1	62.5	<0.05 *	0.083

ES = effect size; ** Significant differences (*p* < 0.01); * Significant differences (*p* < 0.05).

**Table 4:** Differences in the equipment and methodology and communication categories according type of venue.

Category	Item	Total (n = 655)	Public Facilities (n = 188)	Private Facilities (n = 467)		
		%	%	%	*p*	ES
Equipment and methodology	Shared equipment restrictions	85.8	88.8	84.6	0.16	0.06
	Equipment disinfection	74.5	80.3	72.7	<0.05 *	0.09
	Different balls	15.1	18.6	13.7	0.11	0.06
	Drills/Methodology adaptations	94.5	96.3	93.8	0.21	0.05
Communication	Measures	96.6	97.3	96.4	0.53	0.03
	Players’ record	85.8	90.4	83.9	<0.05 *	0.08
	Consent	75.4	79.3	73.9	0.15	0.06
	Online payment and bookings	60.2	59.0	60.6	0.71	0.01

ES = effect size; * Significant differences (*p* < 0.05).

## Data Availability

The data presented in this study are available on request from the corresponding author. The data are not publicly available due to privacy.
